# Prevalence and the evaluation of culture, wet mount, and ELISA methods for the diagnosis of *Trichomonas vaginalis* infection among Ghanaian women using urine and vaginal specimens

**DOI:** 10.1186/s41182-019-0162-9

**Published:** 2019-05-16

**Authors:** Collins Adjei, Richard Boateng, Albert Dompreh, Bismark Okyere, Eddie-Williams Owiredu

**Affiliations:** 1 Department of Clinical Microbiology, School of Medical Sciences, College of Health Sciences, Kwame Nkrumah University of Science and Technology, Kumasi, Ghana; 2 Department of Clinical Microbiology, Komfo Anokye Teaching Hospital, Kumasi, Ghana; 3 Department of Molecular Medicine, School of Medical Sciences, College of Health Sciences, Kwame Nkrumah University of Science and Technology, Kumasi, Ghana

**Keywords:** *Trichomonas vaginalis*, Culture, Wet mount, ELISA

## Abstract

**Background:**

The services of most clinical laboratories in Africa regarding the diagnosis of *Trichomonas vaginalis* are largely dependent on the urine direct wet-mount method. However, the exclusive use of urine-based detection may not be appropriate. The culture method is considered the “gold standard” for the diagnosis of *T. vaginalis*. However, this method has a relatively longer turn-around time and is limited by non-viable organisms in the specimen. This study assessed the prevalence of *T. vaginalis* and its associated risk factors and evaluated its diagnosis using urine and vaginal samples from symptomatic female out-patients by culture, direct wet-mount, and ELISA method respectively.

**Methods:**

This cross-sectional study was conducted at the Obstetrics and Gynaecology department of the Manhyia District hospital (MDH) and Komfo Anokye Teaching Hospital (KATH), Ghana. Ghanaian sexually active female adults between the ages of 18 and 50 years old were recruited for this study. Vaginal (HVS) and urine samples were collected from each participant, and *T. vaginalis* infection was assessed based on culture, direct wet mount, and ELISA methods.

**Results:**

The prevalence of *T. vaginalis* infection based on the ELISA method, HVS culture, and HVS wet mount were 7.2%, 5.0%, and 1.7%, respectively. Urine culture presented with a 0.6% prevalence rate while urine direct wet mount detected no positive case. There was no statistically significant association between demographic and clinical characteristics and *T. vaginalis* infection, except for subjects presenting with abdominal pain [OR = 5.42, 95% CI (1.35–21.73), *p* = 0.017]. Using HVS culture as the reference, ELISA performed best compared to the other methods assessed in this study, presenting with the highest sensitivity [88.9%, 95% CI (54.0–99.8)], specificity [97.1%, 95% CI (93.1–98.9)], AUC (93.0%), and accuracy (96.7%).

**Conclusion:**

The prevalence of *T. vaginalis* infection is high among women in Ghana. With the exception of abdominal pain, there is no significant association between demographic and clinical characteristics and *T. vaginalis* infection. In the event where the culture method is unavailable or when rapid diagnosis is required, antigenic detection using ELISA is the most accurate for the diagnosis of *T. vaginalis* infection in women compared to urine wet-mount/culture and the HVS wet-mount method.

## Background

Trichomoniasis, an infectious disease which mostly occurs in women of reproductive age, is increasing globally. It accounted for 59.7 million new cases of infection in 2008, with 42.8 million infections at any point in time [1]. Currently, the WHO reports 143 million new infections annually [2]. In Africa, a prevalence of 25.64% has been reported [3].

*Trichomonas vaginalis*, a parasitic protozoan, is the causative agent for trichomoniasis. It infects the urogenital tract and has been associated with urethritis, vaginitis, cervicitis, pelvic inflammatory disease, and tubal infertility [4–7]. Recently, *T. vaginalis* has been shown to facilitate the acquisition of HIV [8] as well as cancer [9, 10], preterm delivery, and low birth weight [11].

Due to the several detrimental effects associated with the infection, accuracy and precision in clinical diagnosis is essential to reduce the risk of transmission and morbidity. Direct wet-mount microscopic examination is the most used method for *T. vaginalis* diagnosis, especially in low-and middle-income countries such as Ghana because it is simple and inexpensive [12, 13]. However, direct wet-mount microscopy has low sensitivity and is subject to diagnostic biases as a result of its dependence on operator’s experience and viability of the organism in the specimen [14, 15]. The introduction of staining to the direct microscopic method attempted to solve these problems, but reports indicate no increase in sensitivity compared with the unstained direct wet mount method [16].

Cultivation of *T. vaginalis* is more sensitive and valuable especially when the amount of inoculum is few [17]. As such, the culture technique is considered the “gold standard” for the diagnosis of *T. vaginalis* [18]. Nonetheless, this method is limited in the event of non-viable organisms in specimen [16]. Furthermore, in addition to the relatively longer turn-around time of 2–7 days, not many clinical laboratories in Africa are equipped to accurately perform the culture-based detection method. Recently, the sensitivity of *T. vaginalis* diagnosis has been greatly improved by the use of nucleic acid amplification techniques [15]. However, this technique is expensive and not readily available in many resource-limited settings. As a result, serological methods using monoclonal antibodies have been advocated for detection of *T. vaginalis*. These include latex agglutination, immunofluorescence, enzyme-linked immunosorbent assay (ELISA), and lateral flow techniques [16, 19]. Nevertheless, these serological techniques may be technically demanding. As such, each technique has its own merits and demerits. In Ghana, however, services of most clinical laboratories regarding the diagnosis of *T. vaginalis* are limited to the direct wet-mount microscopic technique using urine as the specimen. Nonetheless, report indicates that exclusive use of urine-based detection of *T. vaginalis* may not be appropriate [14].

This study assessed the prevalence of *T. vaginalis* and its associated risk factors and evaluated its diagnosis using urine and vaginal samples from female out-patients by the direct wet-mount, culture, and ELISA technique, respectively. The finding of this study would equip health service providers with limited resources, the information on which techniques may work best to ensure informed and accurate clinical decisions in this era of evidence-based medicine.

## Materials and methods

### Study design/area

This cross-sectional study was conducted between December, 2016, and September, 2017, at the Obstetrics and Gynaecology department of the Manhyia District hospital (MDH) and Komfo Anokye Teaching Hospital (KATH), both in Kumasi, Ghana. Kumasi has a projected population of 4,780,380 individuals, accounting for 19.4% of Ghana’s total population. The entire Manhyia district is served by MDH, and KATH is the second largest hospital in Ghana, serving patients from the upper part of the country [20].

### Study population

The sample size for this study was calculated using the Raosoft sample size calculator [21]. At 95% confidence level, 7% margin of error, and a response distribution of 50%, a total of 180 symptomatic Ghanaian sexually active female adults aged 18–50 years were recruited for the study.

### Inclusion and exclusion criteria

This study included only symptomatic female patients. Patients attending the out-patient clinic were approached, and the study objectives were explained to them. Subjects who agree to participate in the study provided written informed consent. Included participants were non-pregnant, were not menstruating, and have not had sexual intercourse at least 3 days prior to sampling. Patients who were unwilling to participate and those who had been on antiprotozoal, antibiotics, or steroids for the past 2 weeks were excluded from the study.

### Questionnaire administration and clinical data extraction

A validated questionnaire, designed by reviewing previous studies of similar objectives and adjusted to suit our study objective, was used to obtain relevant socio-demographic and clinical data from each respondent.

### Sample collection and laboratory analysis

Two (2) consecutive vaginal swab samples were collected from the posterior fornix of the vagina using sterile cotton wool swab sticks by specialised female laboratory scientists. The first sample was transferred into 1 ml of 0.85% sterile normal saline and mixed thoroughly. A drop of the mixture was placed on a clean, grease-free microscope slide, covered with a coverslip and observed under light microscope using × 10 objective lens for motile trichomonads, followed by confirmation with × 40 objective lens. In order to increase the chances of recovering the *T. vaginalis*, three slides were prepared for each specimen. Due to the delicate nature of the organism outside its normal vaginal environment, all microscopic examinations were performed within 30 min of sample collection. Specimens with the presence of *T. vaginalis* trophozoites (one or more motile trichomonads) were considered positive by vaginal sample (HVS) wet-mount method. The remainder of the first sample was stored at − 20 °C until *T. vaginalis* antigen testing by ELISA method. The second vaginal swab was inoculated into Kupferberg culture medium (HIMEDIA Laboratories, Mumbai, India) after collection and incubated at 37 °C, with daily microscopic examination for the presence of *T. vaginalis* trophozoites. Negative specimens were continually incubated in the same condition until the 7th day after the initial inoculation, after which samples with no trophozoites upon microscopic examination were considered negative for *T. vaginalis* by HVS culture method.

*T. vaginalis* antigen detection was performed by sandwich ELISA method (Kalon Biological, Guildford, UK) according to the manufacturer’s instructions. Briefly, 50 μL of positive control, negative control, and patient sample were pipetted into respective microtitre wells on a microtitre plate. Enzyme conjugate reagent (100 μL) was added. The content of each well was mixed thoroughly, covered with an adhesive cover, and incubated at 37 °C for 60 min. After incubation, the mixture was aspirated from the wells followed by four washes with the wash solution. Residual wash solution droplets were removed by blotting the microtitre plate onto an absorbent paper. A 100 μL of tetramethylbenzidine (TMB) solution (prepared from 50 μL of chromogen A and 50 μL of chromogen B) was pipetted into each well, mixed gently, and incubated at 37 °C for 15 min. Fifty (50) μL of Stop Solution was added to each well and gently mixed for 30 s to stop the reaction. The absorbance of each well was measured spectrophotometrically at 450 nm using Thermo Electron Multiskan EX plate reader (Shanghai, China).

After collection of vaginal samples, each respondent was asked to provide about 20–40 ml of urine sample. Urine was collected into clean, grease-free, wide-mouth urine containers. The urine was mixed thoroughly; aliquots of 10 ml was prepared and centrifuged at 1000 rpm for 5 min. The supernatant was decanted, and a drop of the sediment was placed on a clean, grease-free microscope slide, covered with a cover slip, and examined following the same protocol used for the vaginal swab specimen. The remaining urine sediment was re-suspended in distilled water, and 100 μl of the suspension was pipetted into Kupferberg culture medium, followed by incubation at 37 °C for 7 days. The protocol for incubation and examination of vaginal swab specimen was similarly applied to the urine specimen. All laboratory analyses were conducted under standard laboratory conditions.

### Statistical analysis

Data processing was done using Microsoft Excel 2016. Statistical analysis and graphical presentation was performed using the R Language for Statistical Computing version 3.5.2 (R Core Team, Vienna, Austria) [22]. Categorical data were presented as frequency (percentages). Univariate logistic regression analysis was used to assess the association between sociodemographic and clinical characteristics and *T. vaginalis* infection. The kappa (ĸ) statistic was used to evaluate the agreement between the various tests used in the study and the receiver operating characteristics (ROC) curve analysis was used to assess the diagnostic performance of the tests. Confidence was set at 95%, and a *p* value < 0.05 was considered statistically significant.

## Results

Table 1 shows the demographic and clinical characteristics of the study population. A higher proportion of the subjects were 21–30 years old (52.2%), were married (65.6%), had basic education (49.4%), had a single sexual partner (85.0%), were unemployed (70.6%), and were living in the urban setting (92.8%). Additionally, a higher proportion of the vaginal samples were whitish in colour (44.4%). Furthermore, the prevalence of vaginal odour, vaginal itchiness, lower abdominal pain, and dysuria were 21.7%, 36.7%, 14.4%, and 7.2%, respectively (Table 1).

**Table 1:** Demographic and clinical characteristics of the study population

Variables	Frequency (*n* = 180)	Percentage
Demographic characteristics		
Age (years)		
< 20	25	13.9
21–30	94	52.2
31–40	50	27.8
41–50	11	6.1
Marital status		
Single	62	34.4
Married	118	65.6
Educational level		
No formal education	9	5.0
Basic	89	49.4
Secondary	50	27.8
Tertiary	32	17.8
Residence		
Rural	13	7.2
Urban	167	92.8
Employment status		
Employed	53	29.4
Unemployed	127	70.6
Number of sexual partners		
0	4	2.2
1	153	85.0
2	19	10.6
3	4	2.2
Clinical characteristics		
Colour of vaginal sample		
Clear	58	32.2
Whitish	80	44.4
Yellowish-green	42	23.3
Vaginal odour	39	21.7
Itchiness	66	36.7
Abdominal pain	26	14.4
Dysuria	13	7.2

The prevalence of *T. vaginalis* infection stratified by type of specimen and test methods applied is shown in Table 2. The prevalence of *T. vaginalis* infection based on the ELISA method, HVS culture, and HVS wet mount were 7.2%, 5.0%, and 1.7%, respectively. Urine culture presented with a 0.6% prevalence rate. However, urine wet mount did not detect any parasite (Table 2).

**Table 2:** The prevalence of *T. vaginalis* infection stratified by type of specimen and test methods applied

Test method	Positive	Negative
ELISA	13 (7.2)	167 (92.8)
Vaginal sample (HVS) culture	9 (5.0)	171 (95.0)
Vaginal sample (HVS) wet mount	3 (1.7)	177 (98.3)
Urine culture	1 (0.6)	179 (99.4)
Urine wet mount	0 (0.0)	180 (100.0)

*ELISA* enzyme-linked immunosorbent assay

Table 3 shows the association between demographic and clinical characteristics and *T. vaginalis* infection. There was no statistically significant association between demographic and clinical characteristics and *T. vaginalis* infection, except women presenting with abdominal pain where an increased odds of *T. vaginalis* infection was observed [OR = 5.42, 95% CI (1.35–21.73), *p* = 0.017] compared to participants who presented with no abdominal pain (Table 3).

**Table 3:** Association between demographic and clinical characteristics and *T. vaginalis* infection

Variables	Negative	Positive	OR (95% CI)	*p* value
Demographic characteristics				
Age (years)				
< 20	25 (100)	0 (0.0)	1	
21–30	88 (93.6)	6 (6.4)	3.75 (0.20–68.76)	0.374
31–40	47 (94.0)	3 (6.0)	3.76 (0.19–75.63)	0.387
41–50	11 (100.0)	0 (0.0)	–	
Marital status				
Single	59 (95.2)	3 (4.8)	1	
Married	112 (94.9)	6 (5.1)	1.05 (0.25–4.36)	0.943
Educational level				
No formal education	8 (88.9)	1 (11.1)	1	
Basic	87 (97.8)	2 (2.2)	0.18 (0.01–2.26)	0.186
Secondary	48 (96.0)	2 (4.0)	0.33 (0.03–4.12)	0.392
Tertiary	28 (87.5)	4 (12.5)	1.14 (0.11–11.72)	0.911
Residence				
Rural	13 (100.0)	0 (0.0)	1	
Urban	158 (94.6)	9 (5.4)	1.62 (0.09–29.34)	0.745
Employment status				
Employed	52 (98.1)	1 (1.9)	1	
Unemployed	119 (93.7)	8 (6.3)	3.50 (0.43–28.67)	0.244
Number of sexual partners				
0	4 (100.0)	0 (0.0)	1	
1	145 (94.8)	8 (5.2)	0.53 (0.03–10.57)	0.675
2	18 (94.7)	1 (5.3)	0.73 (0.03–21.06)	0.854
3	4 (100.0)	0 (0.0)	1.00 (0.02–62.31)	1.000
Clinical characteristics				
Odour				
No	134 (95.0)	7 (5.0)	1	
Yes	37 (94.9)	2 (5.1)	1.03 (0.21–5.19)	0.967
Colour of vaginal sample				
Clear	55 (94.8)	3 (5.2)	1	
Whitish	78 (97.5)	2 (2.5)	0.47 (0.08–2.91)	0.417
Yellowish-green	38 (90.5)	4 (9.5)	1.93 (0.41–9.12)	0.407
Dysuria				
No	159 (95.2)	8 (4.8)	1	
Yes	12 (92.3)	1 (7.7)	1.66 (0.19–14.36)	0.647
Itchiness				
No	108 (94.7)	6 (5.3)	1	
Yes	63 (95.5)	3 (4.5)	0.86 (0.21–3.55)	0.832
Abdominal pain				
No	149 (96.8)	5 (3.2)	1	
Yes	22 (84.6)	4 (15.4)	5.42 (1.35–21.73)	*0.017*

Univariate logistic regression analysis was used to assess the association between demographic and clinical characteristics and *T. vaginalis* infection based on prevalence by the gold standard (HVS culture). *p* values < 0.05 were considered statistically significant (*p* values of significant variables in italics)

*OR* odds ratio

**Fig. 1:**
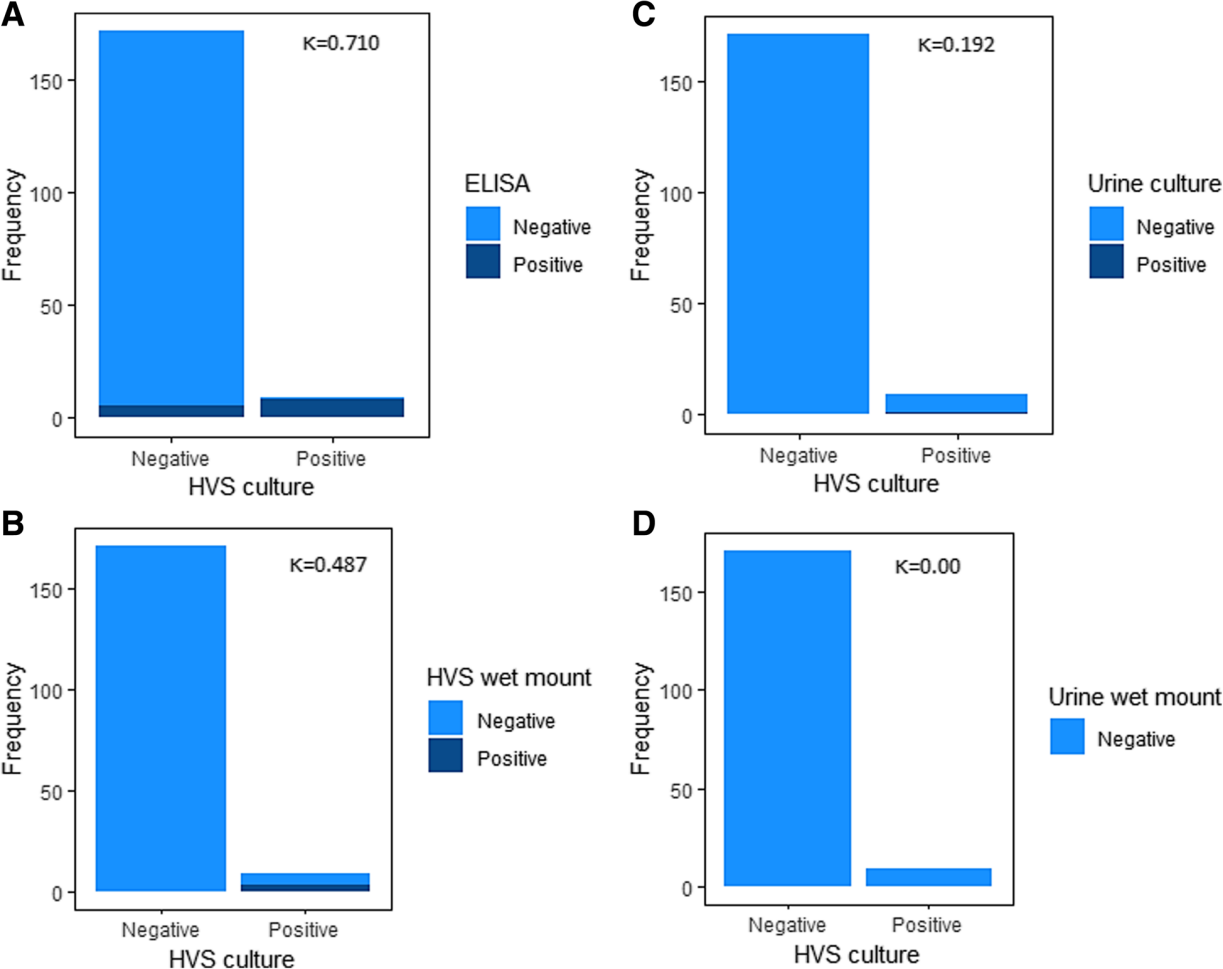
Concordance evaluation between the use of HVS culture and the other tests

As shown in Fig. 1, of the nine (9) cases diagnosed as positive for *T. vaginalis* using the gold standard (HVS culture), eight (8) were identified as positive by the ELISA method with a substantial agreement (*κ* = 0.710) (Fig. 1a), three (3) by the HVS wet mount method with a moderate agreement (*κ* = 0.487) (Fig. 1b), and one (1) by the urine culture method with a slight agreement (*κ* = 0.192) (Fig. 1c). None of the urine samples were positive for *T. vaginalis* using the wet mount method (Fig. 1d).

**Fig. 2:**
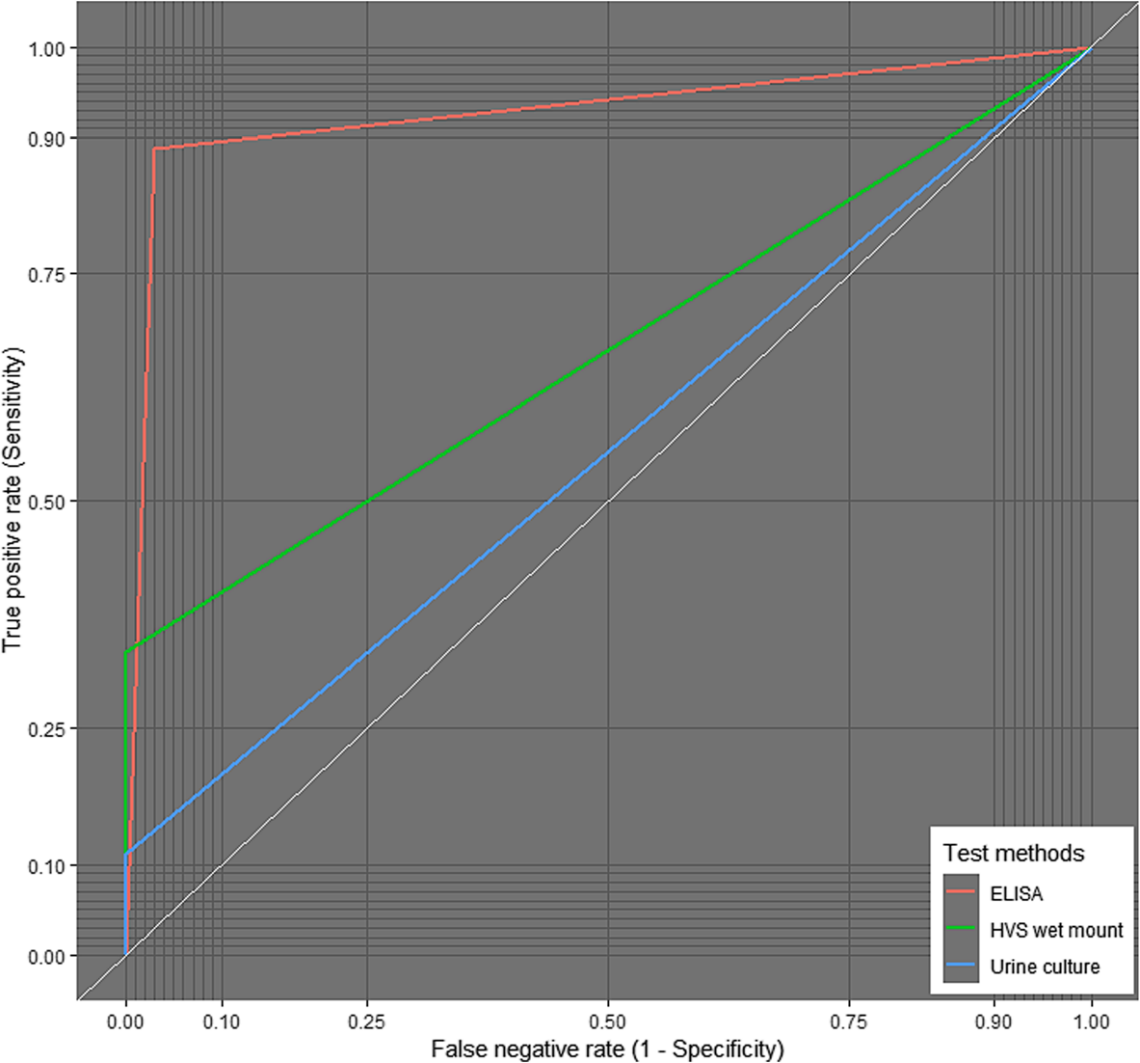
Evaluation of the performance of test methods used

We used the receiver operating characteristics (ROC) curve analysis to evaluate the performance of the test methods assessed in this study using HVS culture as the reference, as shown in Fig. 2 and Table 4. ELISA performed best compared to the other methods used in this study, presenting with the highest sensitivity [88.9%, 95% CI (54.0–99.8)], specificity [97.1%, 95% CI (93.1–98.9)], AUC (93.0%), and accuracy (96.7%). With an accuracy of 96.7%, HVS wet mount presented with a specificity of [100%, 95% CI (97.3–100.0)] but a low sensitivity [33.3%, 95% CI (12.0–64.9)] and AUC (66.7%). Additionally, despite the high specificity [100%, 95% CI (97.2–100.0)] for urine culture, there was a lower sensitivity [11.1%, 95% CI (0.2–45.9)] and accuracy (55.6%) (Table 4).

**Table 4:** Evaluation of the performance of test methods used

Methods*	ELISA	HVS wet mount	Urine culture
Sensitivity (95% CI)	88.9 (54.0–99.8)	33.3 (12.0–64.9)	11.1 (0.2–45.9)
Specificity (95% CI)	97.1 (93.1–98.9)	100 (97.3–100.0)	100 (97.2–100.0)
PPV	61.5	100.0	100.0
NPV	99.4	96.7	95.3
TP	8	3	1
TN	166	171	163
FP	5	0	0
FN	1	6	8
Accuracy (%)	96.7	96.7	95.4
AUC (%)	93.0	66.7	55.6

*PPV* positive predictive value, *NPV* negative predictive value, *TP* true positives, *TN* true negatives, *FP* false positives, *FN* false negatives, *AUC* area under curve

*Urine wet mount has been excluded due to lack of positivity

## Discussion

The prevalence of *T. vaginalis* infection in this present study using the “gold standard” (HVS culture) was 5.0%. This finding is in harmony with the finding of a cross-sectional study by Mahmoud et al. among Egyptian women [16]. In their study, fifty (50) out of 1000 symptomatic female patients were positive for *T. vaginalis* infection. The finding of this present study is also consistent with a cross-sectional study by Al-Saeed et al. who reported a prevalence of 5.4% among a total of 425 vaginal swabs collected from women from Dohok province in Iraq using the culture-based detection method [23]. However, a recent study by Asmah et al. in Ghana reported that 64 of 150 outpatients were positive for *T. vaginalis* [12]. This discrepancy may be due to the fact that Asmah et al. included both males and females in their study while only females were included in this present study. Additionally, they employed polymerase chain reaction (PCR) technique while we did not, which may have underestimated the prevalence rate obtained in this study. Moreover, their study was originally conducted in 2014, and increasing level of knowledge and awareness of the infection may have resulted in the diminution of the prevalence rate. Furthermore, geographical dissimilarities may be partly involved in the differences in prevalence rates because their study was conducted in southern Ghana while this current study took place in northern Ghana.

Another finding of this study is that there was no significant association between demographic and clinical characteristics with *T. vaginalis* infection, except for subjects presenting with abdominal pain, where an increased odds of *T. vaginalis* infection was observed compared with participants who presented with no abdominal pain. Coherent with this finding is a cross-sectional study by Madhivanan et al. among 200 non-pregnant female patients in Egypt [24]. Madhivanan et al. reported no significant association between potential risk factors and *T. vaginalis* infection based on vaginal sample culture using InPouch TV culture kit, with the exception of patients complaining of either dysuria, dyspareunia, or abdominal pain. This finding is also consistent, in part, with a study by Fernando et al. in Sri Lanka [25] among female clinic attendees aged 15–60 years. They reported no significant association between demographic and clinical characteristics and *T. vaginalis* infection based on vaginal sample culture. However, the association between demographic and clinical characteristics with *T. vaginalis* infection remains debatable as there have been reports of significant association with age, ethnicity, and education [26–28]. Verteramo et al. [26] and Helms et al. [27] found a significant associations between *T. vaginalis* infection with older age and low level of education among women in Italy and USA respectively using the modified Diamond’s medium. Another study by Sutton et al. [28] among reproductive-age women in the USA reported that ethnicity, being born in the USA, increasing age, and lower educational level were associated with increased risk of *T. vaginalis* infection based on PCR. Nonetheless, it is worthy of note that, these studies employed more sensitive methods for the detection of *T. vaginalis*. A more sensitive method results in the identification of higher number of positive cases, which may have been missed in this present study, consequently influencing risk associations.

The symptoms of *T. vaginalis* infection are non-specific. As such, diagnosis largely depends on the use of clinical laboratory techniques. Currently, the most commonly used laboratory technique for *T. vaginalis* diagnosis, especially in Africa, is the wet mount method because it is simple and inexpensive [13]. In this present study, direct wet mount using urine specimen did not detect any positive case of *T. vaginalis*. Urine culture, on the other hand, resulted in a 0.6% prevalence rate with a specificity of 100%, sensitivity of 11.1%, and accuracy of 55.6% whereas direct wet mount examination of vaginal specimen resulted in a prevalence of 1.7% [specificity = 100%, sensitivity = 33.3%, and AUC = 66.7%], suggesting that the exclusive use of urine-based detection of *T. vaginalis* may not be appropriate. This finding is in unison with a study by Lawing et al. in USA [14] and Patil et al. in India [29].

The prevalence of *T. vaginalis* infection was highest with ELISA-based antigenic detection method (7.2%). Using the “gold standard” as the reference, the use of ELISA presented with a substantial concordance with the vaginal sample culture method (*κ* = 0.710), with vaginal sample wet mount and urine culture methods presenting with moderate (*κ* = 0.487) and slight agreement (*κ* = 0.192) respectively. In order to evaluate the performance of each test method in diagnosing *T. vaginalis* infection, we employed the receiver operating characteristics (ROC) curve analysis with reference to the gold standard (vaginal sample culture). The ELISA method performed best compared to the other methods, presenting with the highest sensitivity [88.9%, 95% CI (54.0–99.8)], specificity [97.1%, 95% CI (93.1–98.9)], AUC (93.0%), and accuracy (96.7%). This suggest that antigenic detection using ELISA-based method may be used as a surrogate to HVS culture, for accurate diagnosis of *T. vaginalis* infection in women in the event where the culture method is unavailable or when rapid diagnosis is required. Nonetheless, it is worthy of note that there is a false-negative and false-positive rate of 1.1% and 2.9% respectively when the ELISA method was used compared with the vaginal sample culture method; hence, results from the ELISA method should be interpreted with caution. This incongruity may be partly associated with the detection of nonpathogenic trichomonads such as *Pentatrichomonas hominis* [30], probably due to cross-contamination between the anorectal and cervico-vaginal sites, by the ELISA method possibly due to cross-reactivity [31, 32]. Alternatively, the disparity may be linked to the possibility of non-viable *T. vaginalis* in the culture specimen. This is because the culture-based method is chiefly dependent on the viability of the organisms and the presence of non-viable organism will result in a negative culture result [16].

This study is however limited by the fact that we did not perform the more sensitive polymerase chain reaction (PCR) method and this may have underestimated the overall prevalence of the infection.

## Conclusions

The prevalence of *T. vaginalis* infection is high among women in Ghana. With the exception of abdominal pain, there is no significant association between demographic and clinical characteristics and *T. vaginalis* infection. We, thus, recommend increasing the health awareness of females to undertake regular check-ups, especially when they experience abdominal pains. In the event where the culture method is unavailable or when rapid diagnosis is required, antigenic detection using ELISA is the most accurate for the diagnosis of *T. vaginalis* infection in women compared to urine wet-mount/culture and the HVS wet-mount method.

## Abbreviations

ELISA: Enzyme-linked immunosorbent assay
HVS: High vaginal swab
KATH: Komfo Anokye Teaching Hospital
MDH: Manhyia District hospital
